# Dynamic evolution and phylogenomic analysis of the chloroplast genome in Schisandraceae

**DOI:** 10.1038/s41598-018-27453-7

**Published:** 2018-06-18

**Authors:** Bin Li, Yongqi Zheng

**Affiliations:** 1 State Key Laboratory of Tree Genetics and Breeding, Chinese Academy of Forestry, Beijing, China; 2 Research Institute of Forestry, Chinese Academy of Forestry, Beijing, China; 3 Key Laboratory of Tree Breeding and Cultivation of State Forestry Administration, Chinese Academy of Forestry, Beijing, China

## Abstract

Chloroplast genomes of plants are highly conserved in both gene order and gene content, are maternally inherited, and have a lower rate of evolution. Chloroplast genomes are considered to be good models for testing lineage-specific molecular evolution. In this study, we use Schisandraceae as an example to generate insights into the overall evolutionary dynamics in chloroplast genomes and to establish the phylogenetic relationship of Schisandraceae based on chloroplast genome data using phylogenomic analysis. By comparing three Schisandraceae chloroplast genomes, we demonstrate that the gene order, gene content, and length of chloroplast genomes in Schisandraceae are highly conserved but experience dynamic evolution among species. The number of repeat variations were detected, and the Schisandraceae chloroplast genome was revealed as unusual in having a 10 kb contraction of the IR due to the genome size variations compared with other angiosperms. Phylogenomic analysis based on 82 protein-coding genes from 66 plant taxa clearly elucidated that Schisandraceae is a sister to a clade that includes magnoliids, monocots, and eudicots within angiosperms. As to genus relationships within Schisandraceae, *Kadsura* and *Schisandra* formed a monophyletic clade which was sister to *Illicium*.

## Introduction

Chloroplasts are the photosynthetic organelle that provides energy for plants. The chloroplast has its own genome. In angiosperms, most chloroplast genomes are composed of circular DNA molecules ranging from 120 to 160 kb in length and have a quadripartite organization consisting of two copies of inverted repeats (IRs) of approximately 20–28 kb in size, which divide the rest of chloroplast genome into an 80–90 kb large single copy (LSC) region and a 16–27 kb small single copy (SSC) region. Additionally, the chloroplast genome encodes approximately 114 genes, including four ribosomal RNA (rRNAs), 30 transfer RNA (tRNAs), and approximately 80 unique proteins. Chloroplast protein-coding genes are involved in major functions, which include components of the photosynthetic machinery (such as photosystem I (PSI), photosystem II (PSII), the cytochrome b6/f complex, and the ATP synthase), transcription, and translation.

In general, the chloroplast genome has conserved genome structure, gene content and gene order in most angiosperm plants^1,2^. However, structural rearrangements, gene loss, IR expansion and inversion occur in certain lineages. In parasitic plants, pseudogenization, gene deletions, and intron losses commonly occur during chloroplast genome evolution^3^. Some angiosperm plant groups are amenable to large-scale rearrangements, include Campanulaceae^4–6^, Geraniaceae^7–9^ and some legume family species^10^. A pair of large IR could stabilize the chloroplast genome against major structural rearrangements^11,12^. Extensions or contractions of IR regions, gene loss and intron loss also commonly occur during chloroplast genome evolution in angiosperms^13,14^.

In the chloroplast genome, microstructural mutations such as indels, small inversions, and inverted repeats have provided valuable resources to research genome evolution among plants^15^. Additionally, the chloroplast genome contains more repeated sequences, including simple sequence repeats (SSR), short tandem repeats (STR), homopolymeric repeats, and long repeats, which are assumed to have originated from different mechanisms such as gene conversion, intramolecular recombination, and slipped-strand mispairing (SSM)^16^. Repeated sequences are also the main resources for genomic events of duplication, deletion, and rearrangement in chloroplast genomes^15,17^.

Due to maternal inheritance and the rate of evolution, chloroplast genome sequences have long been a focus of research in plant phylogeographic and molecular evolution, as well as phylogenetic, phylogenomic, and genome evolution^14^. As a result of these characteristics, chloroplast genomes are considered good models for testing lineage-specific molecular evolution. For example, in recent years, the complete chloroplast genome as a super-barcode has gained popularity because it provides more information leading to greatly increased resolution at lower plant taxonomic levels^18,19^. SNP and indels were other particularly informative for population and biogeography studies^20^. The development of next-generation sequencing (NGS) and also the third-generation sequencer have provided scientists with faster and less expensive approaches to sequence chloroplast genomes^21^. Schisandraceae is a small family of the order Austrobaileyales consisting of three genera: *Schisandra* Michx. with approximately 25 species, *Kadsura* Kaempf. ex Juss. with approximately 22 species, and *Illicium* L. with approximately 42 species^22^. The majority of Schisandraceae species are distributed in temperate and subtropical forests in Southeast Asia and North America^22,23^. Several species of Schisandraceae have been used in traditional Chinese medicine for many years for the purposes of increasing physical working capacity, relieving pain, and treating skin inflammation^24,25^.

The classification systems before APG II segregated the genus *Illicium* as a distinct family, Illiciaceae, and *Schisandra* and *Kadsura*, in the family Schisandraceae^23,26,27^. In addition, the infra-generic classifications in Schisandraceae are still unstable, while molecular phylogenetic analyses concluded that neither *Schisandra* nor *Kadsura* is monophyletic^24^. Therefore, DNA markers with higher resolution are in need for better determining the unresolved lineages in Schisandraceae. Schisandraceae is one of the earliest diverging lineages in angiosperms, however its chloroplast genome evolved in a unique manner to have a 10 kb contraction of the IR. Previous studies had already discovered this phenomenon in genus *Illicium* and *Schisandra*^28,29^. Whether this pattern stays stable in the family needs further examination.

In the present study, we reconstructed the whole chloroplast genome of *Kadsura coccinea* by using next-generation sequencing and further integrated the available *Illicium* and *Schisandra* chloroplast genomes of Schisandraceae. Hence, every genus of Schisandraceae has its representative species present in this study. The objectives of this study were (1) to establish and characterize the organization of the complete chloroplast genome of *Kadsura coccinea*; (2) to gain in-depth insights into the overall evolutionary dynamics of Schisandraceae chloroplast genomes; and (3) to calibrate the phylogenetic position of Schisandraceae based on phylogenomic analysis.

## Materials and Methods

### Taxon sampling, DNA extraction and sequencing

Fresh leaves of *Kadsura coccinea* were collected from a tree at the Research Institute of Forestry, Chinese Academy of Forestry. The fresh leaves were immediately dried with silica gel before DNA extraction. Total DNA was extracted from approximately 10 g of leaves through an improved method by Li *et al*.^30^. The quality of DNA was determined by a Nanodrop-2000 spectrometer (Nanodrop Technologies, Wilmington, DE, USA) and agarose gel electrophoresis. DNA was randomly fragmented into 400–600 bp using an ultrasonicator. An Illumina paired-end DNA library was constructed using the NEBNext® Ultra™DNA Library Prep Kit following the manufacturer’s instructions. Paired-end sequencing (2 × 150 bp) was carried out on an Illumina HiSeq. 4000 platform.

### Genome assembly and genome annotation

The paired-end reads were qualitatively assessed and initially assembled with SPAdes 3.6.1^31^. Contigs of low sequencing depths were discarded. The remaining contigs may contain the information not only from the chloroplast genome but also from the nuclear genome and the mitochondrial genome. Next, chloroplast genome sequence contigs were selected by performing a BLAST search with default parameters using the *Schisandra chinensis* chloroplast genome sequence as a reference (GenBank accession number: KU362793)^32^. Then, the selected contigs were assembled with Sequencher 5.4.5 (Gene Codes, Ann Arbor, MI). The gaps between the plastomic contigs or ambiguous nucleotides were closed by obtaining amplicons with specific primers and directly sequencing the amplicons. The four junctions between the inverted repeats (IRs) and small single copy (SSC)/large single copy (LSC) regions were confirmed with PCR-based product sequencing^2^.

Chloroplast genome annotation was performed with Plann^33^ using the *Schisandra chinensis* reference sequence from GenBank. A chloroplast genome map was drawn using Genome Vx software^34^. The complete chloroplast genome sequence was deposited in GenBank.

### Repeat sequence and SSR element analyses

The size and location of repeat sequences, including forward and palindromic repeats, within the chloroplast genome of *Illicium oligandrum*, *Kadsura coccinea* and *Schisandra chinensis* were identified using REPuter software^35^. The repeats were identified according to the following conditions: (1) hamming distance of 3, (2) sequence identity ≥90%, and (3) minimum repeat size ≥30 bp. Tandem repeats were identified using web-based Tandem Repeats Finder (https://tandem.bu.edu/trf/trf.html), with 2, 7, and 7 set for the alignment parameters match, mismatch, and indel, respectively. SSRs in the chloroplast genome were detected using MISA (MIcroSAtellite; http://pgrc.ipk-gatersleben.de/misa) with the parameters set at >10 for mononucleotide, >5 for dinucleotide, >4 for trinucleotide, and >3 for tetranucleotide, pentanucleotide, and hexanucleotide SSRs.

### Comparative genome analysis

Genome structures among the three genera of the Schisandraceae were compared using mVISTA software in Shuffle-LAGAN mode^36^. *Illicium oligandrum* was set as a reference. Subsequently, the nucleotide diversity of the chloroplast genome was conducted based on a sliding window analysis with the DnaSP v5.10 software^37^. The step size was set to 200 base pairs, and the window length was set to 800 base pairs. The chloroplast genome borders of LSC, SSC, and IRs were compared according to their annotations.

### Phylogenetic analyses

To examine the phylogenetic position of Schisandraceae in angiosperms and the relationship among genus in Schisandraceae, 59 complete chloroplast genomes representing the lineages of angiosperms, especially early angiosperms, were downloaded from NCBI Organelle Genome Resource database. GenBank information for all of the chloroplast genomes used for the present phylogenetic analyses can be found in Supplementary Table S2.

The 82 shared protein-coding gene sequences were extracted using a Python script and aligned separately by MAFFT v7^38^. The alignment was manually adjusted, and the specific indels were deleted from the sequences. Phylogenetic trees were reconstructed based on 82 concatenated protein-coding gene sequences by maximum likelihood (ML) and Bayesian inference (BI) methods.

The best-fitting model of sequence evolution was identified with ModelFinder^39^ based on the Akaike Information Criterion (AIC). Maximum likelihood (ML) analysis was performed using the IQ-TREE V1.6.1 software package^40^ with 500 non-parametric bootstrap replicates.

Bayesian inference (BI) was performed with MrBayes 3.2.2^41^. Two independent Markov chain Monte Carlo (MCMC) chains, each with three heated and one cold chain, were run for 5 million generations. Each chain started with a random tree, default priors, and sampling trees every 1,000 generations. The MCMC convergence was assumed when the average standard deviation of split frequencies reached 0.01 or less. The first 25% of trees from all runs were discarded as burn-in, and the remaining trees were used to construct majority-rule consensus trees.

### Accession code

*Kadsura coccinea* chloroplast genome are available in GenBank database (accession number: MH029822).

## Results

### Genome content and organization

A total of approximately 5.2 Gb of 150 bp pair-end reads for *Kadsura coccinea* were obtained from the Illumina paired-end sequencing, and the reads were then trimmed and assembled using the SPAdes assembler pipeline. The de novo assembled contigs were searched against the chloroplast genome sequences of *Schisandra chinensis*; eleven contigs were retained. Gaps between contigs were obtained with amplicons from PCR procedures. The total reads were re-mapped to the chloroplast genome, and correction of the sequences was confirmed. Four junction regions of chloroplast genomes were validated using PCR-based sequencing. The coverage of the chloroplast genome was 1233 X, and the sequence of the chloroplast genome was registered into GenBank with the accession number MH029822.

**Figure 1:**
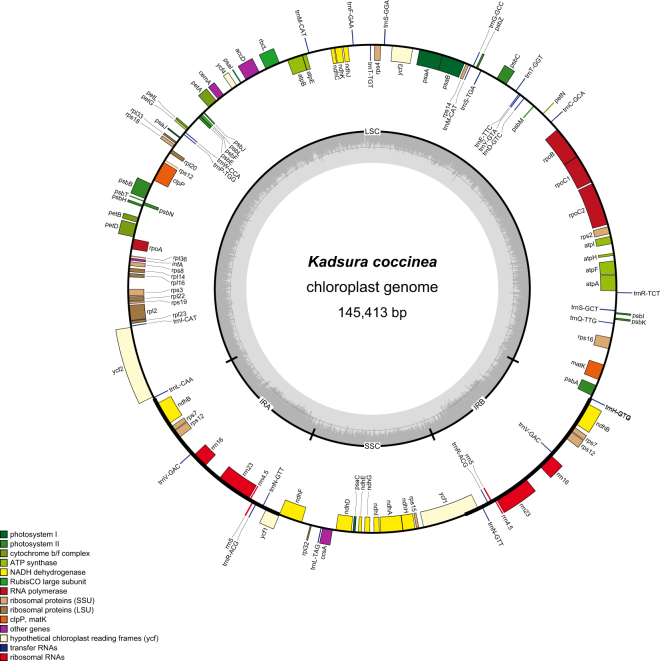
Chloroplast genome map of *Kadsura coccinea*. Genes drawn outside of the circle are transcribed clockwise, while those inside are counterclockwise. Small single copy (SSC), large single copy (LSC), and inverted repeats (IRa, IRb) are indicated. The darker gray represents GC content in the inner circle, conversely the lighter one represents AT content.

Similar to other higher plants, the chloroplast genome of *Kadsura coccinea* had a typical quadripartite structure with two inverted repeats (each 16,536 bp in length) separated by one small single-copy region and one large single-copy region (18,040 and 94,301 bp in length, respectively) (Fig. 1). The chloroplast genome of *Kadsura coccinea* was 145,413 bp in length (Fig. 1). The overall GC content of the chloroplast DNA was 39.7%. The GC content was 38.9%, 35.0%, and 45.5% in the LSC, SSC, and IR regions, respectively. The high GC contents in the IR regions are mainly due to the high GC contents of the four ribosomal RNA (rRNA) genes. Among the representative Schisandraceae species, *Kadsura coccinea* exhibits the smallest genome size compared with the other two chloroplast genomes. The genome of *Illicium oligandrum* (148,553 bp) is approximately 3.1 kb larger than that of *Kadsura coccinea* and 0.8 kb larger than that of *Schisandra chinensis*. The detected sequence length difference is predominantly attributable to the variation in the length of the intergenic spacer regions (Table 1).

**Table 1:** Summary of the complete chloroplast genome characteristics of three species in Schisandraceae.

Species	*Kadsura coccinea*	*Schisandra chinensis*	*Illicium oligandrum*
Total	145,413	147,772	148,553
LSC	94,301	97,351	98,057
IR	16,536	15,058	15,114
SSC	18,040	20,305	20,267
Total	113	113	113
Protein coding genes	79	79	79
rRNA	30	30	30
tRNA	4	4	4
GC%	39.7	39.5	39.1

All three Schisandraceae chloroplast genomes encoded 113 unique genes, including 79 protein-coding genes, 30 tRNA genes, and 4 ribosomal RNA genes. Of these, four protein-coding genes, four rRNA genes, and five tRNA genes were duplicated in the IR regions. There were 18 intron-containing genes (one class I intron in *trnL-UAA* and 17 class II introns), of which two genes, *clpP* and *ycf3*, contained two introns and the rest had only one intron each. In *rps12*, a trans-splicing event was observed, with the 5′ end located in the LSC region and the duplicated 3′ end in the IR region. The *trnK-UUU* gene harbours the largest intron, which contains the *matK* gene.

### Repeat sequence analysis

**Figure 2:**
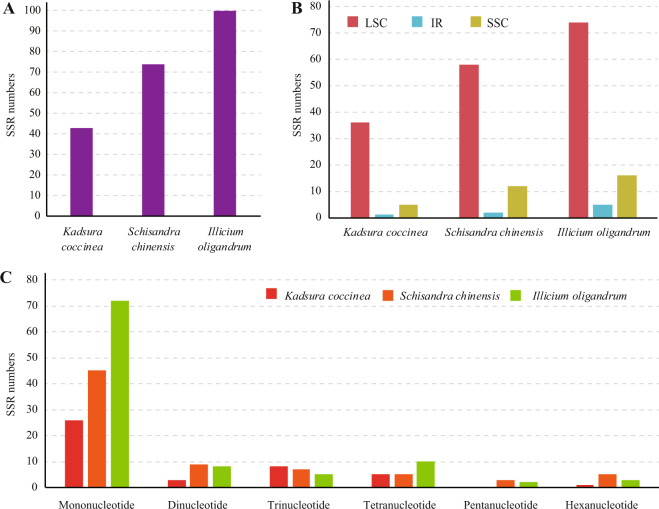
The distribution, type and presence of simple sequence repeats (SSRs) in the three chloroplast genome of Schisandraceae. (**A**) Number of different SSRs types. (**B**) Number of different SSRs in the LSC, SSC, and IR regions. (**C**) Number of identified SSR motifs in different repeat class types.

Considering the role of chloroplast genome SSRs as important phylogenetic markers and valuable resources to indicate genome evolution, we screened and quantified six kinds of repeat patterns in Schisandraceae (Fig. 2). The total number of SSRs identified in the *Kadsura coccinea*, *Schisandra chinensis*, and *Illicium oligandrum* was 43, 74 and 100, respectively. The most abundant SSRs were A or T mononucleotide repeats, which accounted for approximately 60.5%, 56.8% and 69% of the total SSRs in *Kadsura coccinea*, *Schisandra chinensis*, and *Illicium oligandrum*, respectively, while the G or C repeats were rare. The number of penta- and hexanucleotides were slightly less than other repeats, such as di-, tri-, and tetranucleotides. Furthermore, the majority of SSRs of *Kadsura coccinea*, *Schisandra chinensis*, and *Illicium oligandrum* SSRs were located in LSC regions (83.7%, 78.3% and 74.0%, respectively), followed by SSC regions (11.6%, 16.2% and 16.0%, respectively) and IR regions (2.3%, 2.7% and 5.0%, respectively).

**Figure 3:**
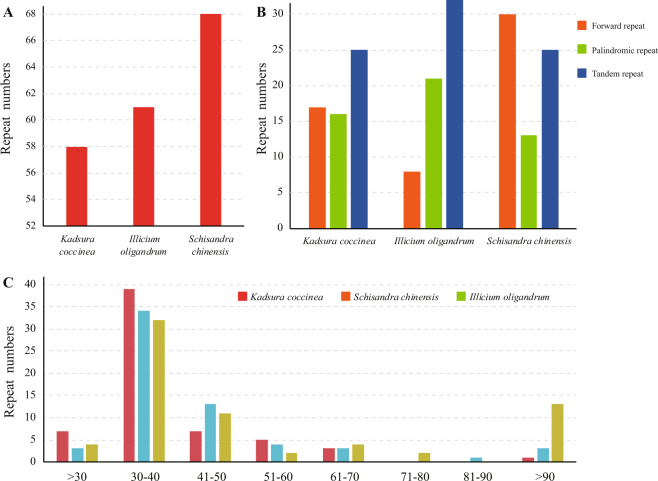
Long repeat sequences in the three chloroplast genome of Schisandraceae. (**A**) Number of repeats. (**B**) Number of different repeats types. (**C**) Sequence length of repeats.

In addition to the SSRs, we employed REPuter and Tandem Repeats Finder to analyse the repeat sequences of the three chloroplast genomes (Fig. 3). The total number of repeats was 58 in *Kadsura coccinea*, 68 in *Schisandra chinensis*, and 61 in *Illicium oligandrum*. *Kadsura coccinea* contained 17 forward repeats, 16 palindrome repeats, and 25 tandem repeats. *Schisandra chinensis* contained 30 forward repeats, 13 palindrome repeats, and 25 tandem repeats, while *Illicium oligandrum* contained 8 forward repeats, 21 palindrome repeats, and 32 tandem repeats. Lengths of 30–40 repeats were the most common (average 56.7%), follow by 41–50 repeats. In addition, the proportions of repeats located in non-coding regions were higher than in coding regions.

### Sequence divergence and divergence hotspot

**Figure 4:**
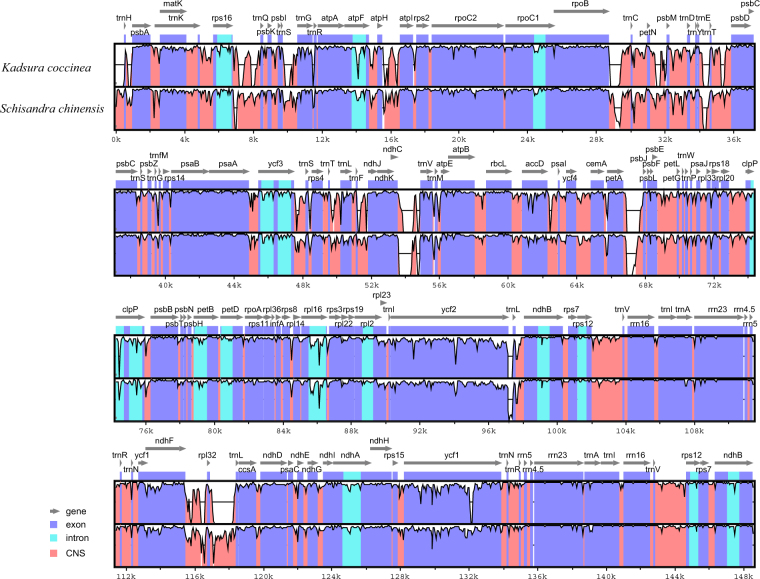
Sequence comparison of the *Illicium oligandrum*, *Kadsura coccinea* and *Schisandra chinensis* chloroplast genomes generated by mVISTA. VISTA based similarity graphical information portraying sequence identity of *Illicium oligandrum* with reference *A*. *indica* chloroplast genomes. Grey arrows above the alignment indicate the orientation of genes. Purple bars represent exons, blue ones represent introns, and pink ones represent non-coding sequences (CNS). A cut-off of 50% identity was used for the plots. The Y-scale axis represents the percent identity within 50–100%. Dashed rectangles indicate highly divergent regions of *Illicium oligandrum* compared with *Kadsura coccinea* and *Schisandra chinensis*.

The mVISTA and DnaSP program were employed to analyse the overall sequence identity at the chloroplast genome level and to detect the divergent regions in the Schisandraceae chloroplast genome (Figs 4 and S2). The organization of the chloroplast genome among any of the compared genomes revealed a high degree of synteny and gene order conservation, suggesting an evolutionary conservation of these genomes at the genome-scale level. Overall, the results revealed higher divergence in non-coding regions than in coding regions. The coding regions with marked differences include the *ycf1*, *accD* and *ndhF* genes. The highest divergence in non-coding regions was found for *rps16-trnQ, atpF-atpH, petN-psbM, trnT-psbC, ycf2-trnL, rpoB*-*trnC*, *ndhC*-*trnV*, *petA*-*psbJ*, *ndhF-rpl32*, and *rpl32*-*trnL*. Notably, the LSC region and SSC region were more divergent than the IR regions.

### IR expansion and contraction

**Figure 5:**
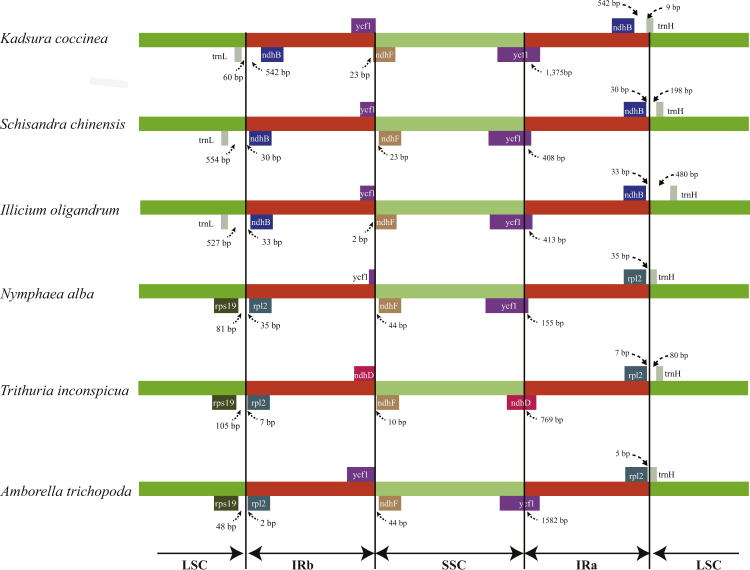
Comparison of the border positions of the LSC, SSC, and IR regions among the six chloroplast genomes of basal angiosperms. Gene names are indicated in boxes, and their lengths in the corresponding regions are displayed above the boxes.

IR expansion and contraction often results in genome size variations among various plant lineages, which can be used to study the phylogenetic classification and the genome evolution among plant lineages^13^. In the present study, the IR boundary regions of three Schisandraceae species and three other early-diverging angiosperm species were compared, and the results showed that the border of the Schisandraceae chloroplast genome was slightly different from that of other genomes (Fig. 5). In Schisandraceae, the boundary was between *trnL-CAA* and *ndhB* on the IRb/LSC side and between *ndhB* and *trnH-GUG* on the IRa/LSC side. The boundary of IRb/LSC occurred between *rps19* and *rpl2* and between *rpl2* and *trnH-GUG* on the IRa/LSC side, with 0 and 80 non-coding nucleotides between these two genes. The IR in Schisandraceae had a 10 kb contraction compared with other lineages.

The IRa/SSC border extended into *ycf1* resulting in a pseudogene in the three Schisandraceae chloroplast genomes that were compared. The length of the *ycf1* pseudogene was 1,375 bp in *Kadsura coccinea*, 408 bp in *Schisandra chinensis*, and 403 bp in *Illicium oligandrum*. Furthermore, *ndhF* deviated from the IRb/SSC in *Schisandra chinensis* by 23 bp. The *trnH-GUG* gene was generally located downstream of the IR_A_/LSC border, and this gene is separated from the IR_B_/LSC border by 9 bp in *Kadsura coccinea*, 198 bp in *Schisandra chinensis*, and 480 bp in *Illicium oligandrum*. Overall, the IR boundary regions varied slightly in the Schisandraceae chloroplast genome.

### Phylogenomic analysis

**Figure 6:**
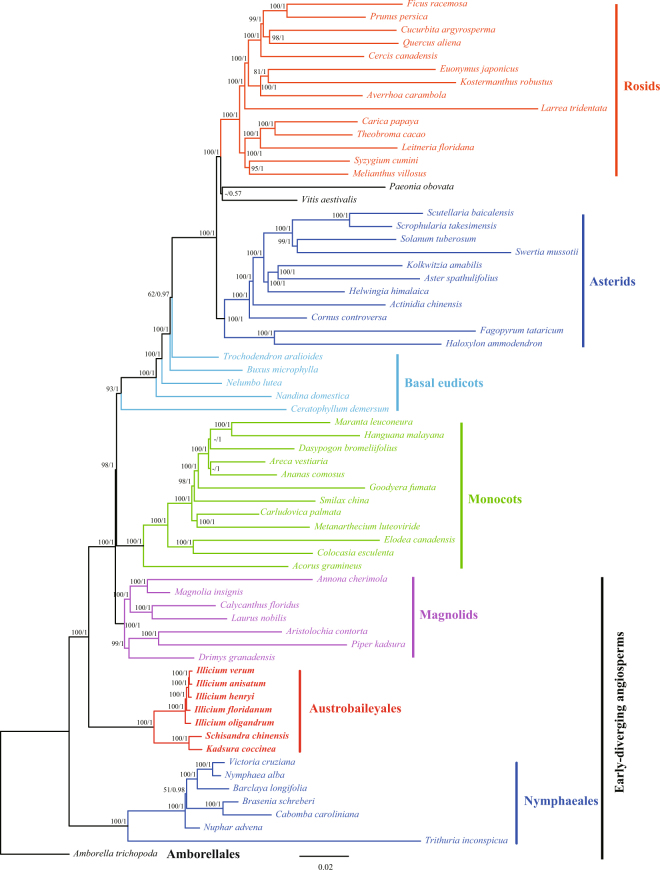
Phylogenetic tree reconstruction of 66 taxa using maximum likelihood and Bayesian inference based on concatenated sequences of 82 genes. ML topology shown with ML bootstrap support value/Bayesian posterior probability given at each node.

Chloroplast genome sequences have been widely used to reconstruct plant phylogenies^42,43^. To examine the phylogenetic position of Schisandraceae within angiosperms and the genus relationship within Schisandraceae, ML and BI methods of phylogenetic analysis were performed based on 82 protein-coding gene datasets from 66 plant taxa, including seven Schisandraceae species. The total alignment was 65,432 bp in length. Both the ML and BI trees had similar phylogenetic topologies, and most nodal support values were high (ML bootstrap support value >95/Bayesian posterior probability >0.99; Figs 6 and S1).

The trees provide support for the following relationships: *Amborella* and Nymphaeales are sisters to the remaining angiosperms; Schisandraceae is sister to a clade that includes magnoliids, monocots, and eudicots; magnoliids and monocots were both monophyletic; Amborellales, Nymphaeales, Austrobaileyales, and magnoliids formed the early-diverging angiosperms; basal eudicots were not monophyletic and *Ceratophyllum* was the sister to the remaining eudicots; rosids and asterids were each monophyletic; and *Vitis* and *Paeonia* were the earliest diverging lineage of rosids.

Schisandraceae was grouped in both ML and BI phylogenetic trees with 100% bootstrap values and 1.0 Bayesian posterior probability. *Kadsura* and *Schisandra* formed monophyly clades and were sisters to *Illicium*. Phylogenetic relationships among the five *Illicium* species were also established using this dataset.

## Discussion

The organization of the Schisandraceae chloroplast genomes was similar to the angiosperm genome except the IR contraction. IR expansion/contraction also represents a highly variable region, which can be used to study molecular classification and the phylogenetic classification of plants. In this study, by comparing the inverted repeat/single copy (IR/SC) boundaries of the four basal angiosperms, we detected a 10 kb IR contraction in Schisandraceae. IR expansion/contraction has occurred multiple times in angiosperms based on the phylogenetic results. In the monocots, expansion of the IR has occurred on the IRa/LSC boundary resulting in a duplicate copy of the *trnH*-GUG gene next to *rps19* at the IRb/LSC boundary. *Adenophora stricta* had a larger IR contraction with eight lost duplication genes compared with other Campanuloid species^5^. Small expansions and contractions of less than 1,000 bp was common in angiosperms, for example Oryza^44^. Three reasons may explain the diversification of IR boundary regions sequences. The first is intramolecular recombination, the second is the presence of multiple repeat sequences, and the third is the indels, which caused a mismatch that resulted in the upstream sequence becoming a single copy^28^.

SSRs are a type of 1–6 bp repeat frequently observed in chloroplast genomes, which can be used to unravel genome polymorphisms and perform population genetics of and across species^45–48^. In this study, the number of SSRs in the three Schisandraceae chloroplast genomes varied. Compared with the other species, the number of SSRs in the *Kadsura coccinea* was approximately the same as those in *Forsythia suspense*^49^ and *Lagerstroemia*^50^ but was two times lower than that in *Illicium oligandrum*. SSR primers for chloroplast genome are transferable across species and genera, because of the chloroplast genome conservation. The SSRs provided molecular markers for studying the genetic diversity and population structure of Schisandraceae species. Repeat sequences provided valuable resources to study genome recombination and rearrangement^8^. About sixty repeats in the Schisandraceae chloroplast genomes were found by REPuter, and this finding was similar in the plant^51^. Most repeat sequences and SSRs were distributed within noncoding regions, and chloroplast genome noncoding regions have been shown to be more variable than coding regions and to play an important role in phylogenetic studies in angiosperms^52^.

The chloroplast genomes are characterized by relatively small size, largely uniparental inheritance, conservation of gene content and order, and high copy number compared to the nuclear genome^53^. Hence, the chloroplast genome sequences have become widely used to resolve phylogenetic relationships among plants. With NGS technology, the chloroplast genome can be efficiently and economically obtained, and sequence data from the chloroplast genome have transformed plant systematics and contributed greatly to the current view of plant relationships^54–62^. A phylogenetic tree was constructed based on 82 protein-coding genes from 66 chloroplast genome sequences, which may represent the major angiosperm clades. ML and BI trees confirmed that Schisandraceae as one of the earliest diverging angiosperm lineages, and the position of the family was just internal to *Amborella* and Nymphaeales. The chloroplast genome data also established the internal relationship of *Illicium* with strong support. Therefore, the chloroplast genome sequence data were effective for inferring the backbone relationships among other family clades of angiosperms, as well as for resolving the phylogenetic relationship of species.

## Electronic supplementary material

Supplementary information
