# Supplementary material for: Dynamic evolution and phylogenomic analysis of the chloroplast genome in Schisandraceae
Source: Sci Rep. 2018 Jun 18;8:9285. doi: 10.1038/s41598-018-27453-7 (PMC6006245; doi:10.1038/s41598-018-27453-7)

Dynamic evolution and phylogenomic analysis of the chloroplast genome in Schisandraceae

Bin Li, Yongqi Zheng*

[1]State Key Laboratory of Tree Genetics and Breeding, Chinese Academy of Forestry, Beijing, China

[2]Research Institute of Forestry, Chinese Academy of Forestry, Beijing, China

[3]Key Laboratory of Tree Breeding and Cultivation of State Forestry Administration, Chinese Academy of Forestry, Beijing, China

* Corresponding author

E-mail: zyq8565@126.com

Table S1. Gene content of the *Kadsura coccinea* chloroplast genome.

Table S2. List of chloroplast genomes sequences used for phylogenetic analysis.

Figure S1. Phylogenetic tree reconstruction of 66 taxa using Bayesian inference methods based on concatenated sequences of 82 genes.

Figure S2. Sliding window analysis of the whole chloroplast genome of three Schisandraceae species (window length: 800bp, step size: 200bp). X-axis: position of the midpoint of a window, Y-axis: nucleotide diversity of each window.

Table S1. Gene content of the *Kadsura coccinea* chloroplast genome.

**Category for genes**	**Group of gene**	**Name of gene**
Photosynthesis related genes	Rubisco	*rbcL*
	Photosystem Ⅰ	*psaA,psaB,psaC,psaI,psaJ*
	Assembly/stability of photosystem Ⅰ	**ycf3,ycf4*
	Photosystem Ⅱ	*psbA,psbB,psbC,psbD,psbE,psbF,psbH,psbI,psbJ,psbK,psbL,psbM,psbN,psbT,psbZ*
	ATP synthase	*atpA, atpB, atpE, *atpF, atpH, atpI*
	cytochrome b/f compelx	*petA, *petB, *petD, petG, petL, petN*
	cytochrome c synthesis	*ccsA*
	NADPH dehydrogenase	**ndhA, *ndhB, ndhC, ndhD, ndhE, ndhF ,ndhG, ndhH, ndhI, ndhJ, ndhK*
Transcription and translation related genes	transcription	*rpoA, rpoB, *rpoC1, rpoC2*

	ribosomal proteins	rps2, rps3, rps4, rps7, rps8, rps11, *rps12, rps14,rps15, *rps16, rps18, rps19,*rpl2, rpl14, *rpl16, rpl20, rpl22, rpl23, rpl32, rpl33,rpl36
	translation initiation factor	infA
RNA genes	ribosomal RNA	rrn5, rrn4.5, rrn16, rrn23
	transfer RNA	*trnA$_{UGC}$, trnCGCA, trnDGUC, trnEUUC, trnFGAA,trnGGCC, *trnGUCC, trnHGUG, trnICAU, *trnIGAU,*trnKUUU, trnLCAA, *trnLUAA, trnLUAG, trnfMCAUI,trnMCAU, trnNGUU, trnPUGG, trnQUUG,trnRACG, trnRUCU, trnSGCU, trnSGGA, trnSUGA, trnTGGU,trnTUGU, trnVGAC, *trnVUAC, trnWCCA, trnYGUA
Other genes	RNA processing	matK
	carbon metabolism	cemA
	fatty acid synthesis	accD
	proteolysis	*clpP
Genes of unknown function	conserved reading frames	ycf1, ycf2,

Table S2. List of chloroplast genomes sequences used for phylogenetic analysis.

Order	Family	*Species*	GenBank accession number
-	Acoraceae	*Acorus gramineus*	KP099646
-	Araceae	*Colocasia esculenta*	JN105689
-	Arecaceae	*Areca vestiaria*	KT312940
-	Dasypogonaceae	*Dasypogon bromeliifolius*	JX088665
-	Hydrocharitaceae	*Elodea canadensis*	JQ310743
Amborellales	Amborellaceae	*Amborella trichopoda*	NC 005086
Aquifoliales	Helwingiaceae	*Helwingia himalaica*	KX434807
Asparagales	Orchidaceae	*Goodyera fumata*	KJ501999
Asterales	Asteraceae	*Aster spathulifolius*	KF279514
Austrobaileyales	Schisandraceae	*Illicium anisatum*	NC 034703
Austrobaileyales	Schisandraceae	*Illicium floridanum*	NC 034685
Austrobaileyales	Schisandraceae	*Illicium henryi*	NC 034699
Austrobaileyales	Schisandraceae	*Illicium oligandrum*	NC 009600
Austrobaileyales	Schisandraceae	*Illicium verum*	NC 034689
Austrobaileyales	Schisandraceae	*Schisandra chinensis*	NC 034908
Brassicales	Caricaceae	*Carica papaya*	EU431223
Buxales	Buxaceae	*Buxus microphylla*	EF380351
Canellales	Winteraceae	*Drimys granadensis*	NC 008456
Caryophyllales	Chenopodiaceae	*Haloxylon ammodendron*	KF534478
Caryophyllales	Polygonaceae	*Fagopyrum tataricum*	KX085498
Celastrales	Celastraceae	*Euonymus japonicus*	KP189362
Ceratophyllales	Ceratophyllaceae	*Ceratophyllum demersum*	AM712908
Commelinales	Hanguanaceae	*Hanguana malayana*	KT312930
Cornales	Cornaceae	*Cornus controversa*	KU852492
Cucurbitales	Cucurbitaceae	*Cucurbita argyrosperma*	KT898803
Dioscoreales	Nartheciaceae	*Metanarthecium luteoviride*	KT895904
Dipsacales	Caprifoliaceae	*Kolkwitzia amabilis*	KT966716
Ericales	Actinidiaceae	*Actinidia chinensis*	KP297242
Fabales	Fabaceae	*Cercis canadensis*	KF856619
Fagales	Fagaceae	*Quercus aliena*	KP301144
Gentianales	Gentianaceae	*Swertia mussotii*	KU641021
Geraniales	Francoaceae	*Melianthus villosus*	KF017614
Lamiales	Lamiaceae	*Scutellaria baicalensis*	KR233163
Lamiales	Scrophulariaceae	*Scrophularia takesimensis*	KM590983
Laurales	Calycanthaceae	*Calycanthus floridus* var. *glaucus*	NC 004993
Laurales	Lauraceae	*Laurus nobilis*	NC 034700

Liliales	Smilacaceae	*Smilax china*	HM536959
Magnoliales	Annonaceae	*Annona cherimola*	NC 030166
Magnoliales	Magnoliaceae	*Magnolia insignis*	NC 035657
Malpighiales	Chrysobalanaceae	*Kostermanthus robustus*	KX180073
Malvales	Malvaceae	*Theobroma cacao*	HQ244500
Myrtales	Myrtaceae	*Syzygium cumini*	GQ870669
Nymphaeales	Cabombaceae	*Brasenia schreberi*	NC 031343
Nymphaeales	Cabombaceae	*Cabomba caroliniana*	NC 031505
Nymphaeales	Hydatellaceae	*Trithuria inconspicua*	NC 020372
Nymphaeales	Nymphaeaceae	*Barclaya longifolia*	NC 035633
Nymphaeales	Nymphaeaceae	*Nuphar advena*	NC 008788
Nymphaeales	Nymphaeaceae	*Nymphaea alba*	NC 006050
Nymphaeales	Nymphaeaceae	*Victoria cruziana*	NC 035632
Oxalidales	Oxalidaceae	*Averrhoa carambola*	KU569488
Pandanales	Cyclanthaceae	*Carludovica palmata*	KP462882
Piperales	Aristolochiaceae	*Aristolochia contorta*	NC 036152
Piperales	Piperaceae	*Piper kadsura*	NC 027941
Poales	Bromeliaceae	*Ananas comosus*	AP014632
Proteales	Nelumbonaceae	*Nelumbo lutea*	FJ754269
Ranunculales	Berberidaceae	*Nandina domestica*	DQ923117
Rosales	Moraceae	*Ficus racemosa*	KT368151
Rosales	Rosaceae	*Prunus persica*	HQ336405
Sapindales	Simaroubaceae	*Leitneria floridana*	KT692940
Saxifragales	Paeoniaceae	*Paeonia obovata*	KJ206533
Solanales	Solanaceae	*Solanum tuberosum*	KM489056
Trochodendrales	Trochodendraceae	*Trochodendron aralioides*	KC608753
Vitales	Vitaceae	*Vitis aestivalis*	KT997470
Zingiberales	Marantaceae	*Maranta leuconeura*	KF601571
Zygophyllales	Zygophyllaceae	*Larrea tridentata*	KT272174

Figure S1. Phylogenetic tree reconstruction of 66 taxa using Bayesian inference methods based on concatenated sequences of 82 genes.

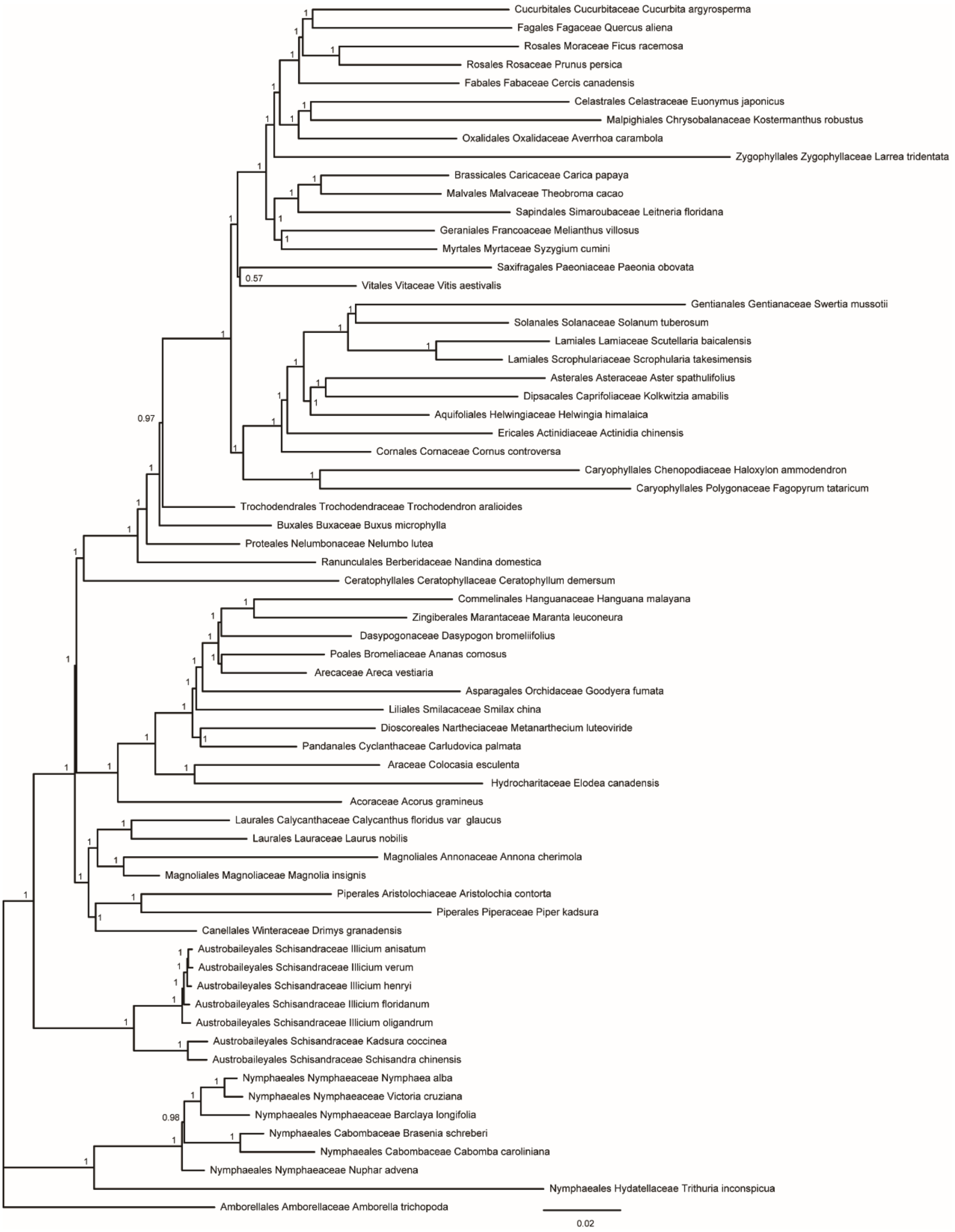

Figure S2. Sliding window analysis of the whole chloroplast genome of three Schisandraceae species (window length: 800bp, step size: 200bp). X-axis: position of the midpoint of a window, Y-axis: nucleotide diversity of each window.

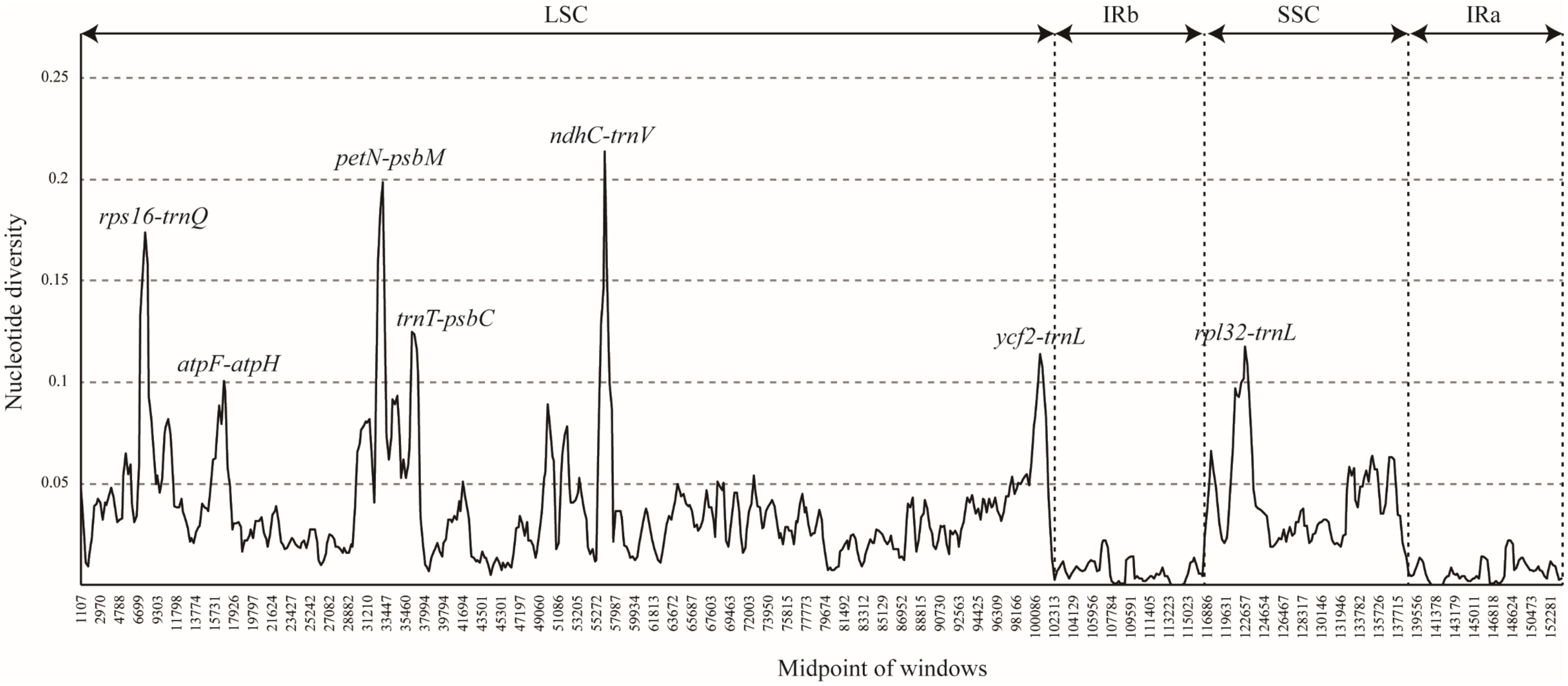

Supplement: Supplementary file 1 — Supplementary information [file 41598_2018_27453_MOESM1_ESM.pdf]
